# Enhancing Output Power of a Cantilever-Based Flapping Airflow Energy Harvester Using External Mechanical Interventions

**DOI:** 10.3390/s19071499

**Published:** 2019-03-28

**Authors:** Liuqing Wang, Dibin Zhu

**Affiliations:** College of Mathematics, Engineering and Physical Sciences, University of Exeter, Exeter EX4 4QF, UK; l.wang4@exeter.ac.uk

**Keywords:** energy harvesting, flapping, galloping, mechanical interventions, magnetic excitation

## Abstract

This paper presents a flapping airflow energy harvester based on oscillations of a horizontal cantilever beam facing the direction of airflow. A wing is attached to the free end of a cantilever beam and a bluff body is placed in front of the wing from where vortex falls off, producing vortices under the wing and driving it to oscillate. An electromagnetic transducer is integrated to convert the flow induced vibration into electrical energy. This flapping energy harvester, however, may stop oscillating or vibrate in the second mode under high electrical damping, and thus may be unable to achieve its optimum performance. Simple yet effective mechanical interventions can be applied to the harvester to enhance its power output, i.e., to increase flow velocity and to apply external magnetic interaction. The effect of airflow velocities on output power was investigated experimentally and the results show that the energy harvester scavenges more power in airflow at higher Reynolds numbers (higher flow velocity at Re < 24,000). The external magnetic excitation is achieved though placing one magnet to the wing and another one above the wing to induce a repelling force, aiding the beam to oscillate in high electrical damping. Experimental results show that the power output can be enhanced by 30% when the magnet interaction is properly integrated.

## 1. Introduction

Flapping or galloping energy harvesters are promising solutions to scavenge energy from airflow via vortex induced vibrations (VIV). Reported flapping energy harvesters are generally designed based on flapping foils or a clamped beam with a bluff body. Numerous work has been done to improve its configuration, effectiveness and efficiency.

Zhu and Peng [1] modelled a flow energy harvester with a flapping foil mounted on a spring-damper (parallel connected) base, using a 2D thin-plate model and a 3D boundary-element model. Based on the optimized configurations, with a 30${}^{\circ }$ pitching amplitude, a 10 m long foil with a 1 m chord is able to generate 8 kW power in a 2 m·s${}^{-1}$ water flow. The unstable modes in wake was numerically associated with energy harvesting efficiency based on heaving/pitching foils [2] and a working frequency close to the most unstable mode achieved the maximum efficiency. A judicious deformation of the camber line was proved to be effective to increase the relative aerodynamic efficiency by 15% [3]. Non-sinusoidal pitching motions of a semi-active flapping foil were investigated and found effective in improving the performance of the system only at small pitching amplitudes [4]. Wu et al. [5] numerically investigated three techniques to enhance the power extraction based a flapping foil. By placing the foil near a solid wall and between two parallel ones, the power generation efficiency has improved respectively from 7.63% to 18.06% and from 14.67% to 27.69% [5]. The second technique is to add a flexible tail to the flapping foil. According to simulation results, a low mass tail with high flexibility has the potential for power increase [6]. Two auxiliary wings were added to the main flapping foil and they numerically studied the induced vortex interaction and the enhancement of power output by application of additional foils [7].

A galloping energy harvester is usually composed of a clamped beam with a bluff body mounted on its free end. The cross section geometry of a bluff body is an important factor in galloping oscillations [8] and the configurations investigated include cylinder, triangle, pentagon, D-shaped, square and trapezoid [9,10,11]. Bibo and Daqaq [10] developed a piezoelectric galloping harvester and established a universal response relationship between the power output and flow velocity to evaluate the performance of three bluff body configurations—a square section, a semi-circle section and a triangle, among which the square section outperforms the others. In another study of square, trapezoid and triangle cross sections, the harvester with a trapezoid bluff body has power output enhanced by 67% [11]. Modification on a plain square bluff body may also contribute to power extraction. Two fins were added to the leading edge of a square prism, enhancing the power output by 250% compared to the application of a plain square [12]. Two Y-shaped attachments were mounted on the circle bluff body of a piezoelectric wind harvester and enhanced the power output by approximately 300% [13]. Zhao et al. [14] worked on the beam configuration and added an inner beam and two magnets to the outer beam to create a stiffness nonlinearity to help the device to operate at low wind speeds (1 m·s${}^{-1}$) and within wider speed range (1–5 m·s${}^{-1}$). Dai et al. [15] developed a galloping electromagnetic energy harvester, with the bluff body at the free end of the beam substituted by a magnet oscillating in a coil and generating power. The extracted power was up to approximately 0.016 W·m${}^{-1}$ in a 4.0 m·s${}^{-1}$ airflow. Bibo et al. [16] worked on the modelling of a galloping piezoelectric harvester and developed a nonlinear distributed-parameter model to predict its response behaviour under combined galloping and base excitations; they later developed a nondimensional lumped-parameter model of the same harvester under quasi-steady aerodynamics (only base excitations) [17]. Pure resistive circuitry was applied in some galloping energy harvesting studies and its effect on the performance of the device has been discussed [15,18,19]. The electrical load resistance can be properly tuned for an optimum electrical damping to achieve best performance, which, however, may be unachievable [18,19] because the resistive load may also significantly influence the instability of the device [15]. Electrical interventions have been reported [19] to cope with this problem—an inductor was placed in series or in parallel with the load to help achieve the optimal damping.

A similar problem has been encountered in a type of flapping airflow energy harvester [20]. The flapping airflow energy harvester, based on a cantilever beam, may stop oscillating or vibrate in the second mode with the increase of electrical damping, thus being unable to achieve its optimum performance. Mechanical interventions are investigated in this research, in order to aid the oscillation of the harvester in high damping condition. Original design and principles will be presented in the second section. The damping effect on the magnification of oscillation amplitude is then discussed to help understand the mechanical intervention. The effects of airflow velocities and of external magnetic excitation on power output are investigated experimentally with results presented and discussed.

## 2. Original Design and Principles

**Figure 1:**
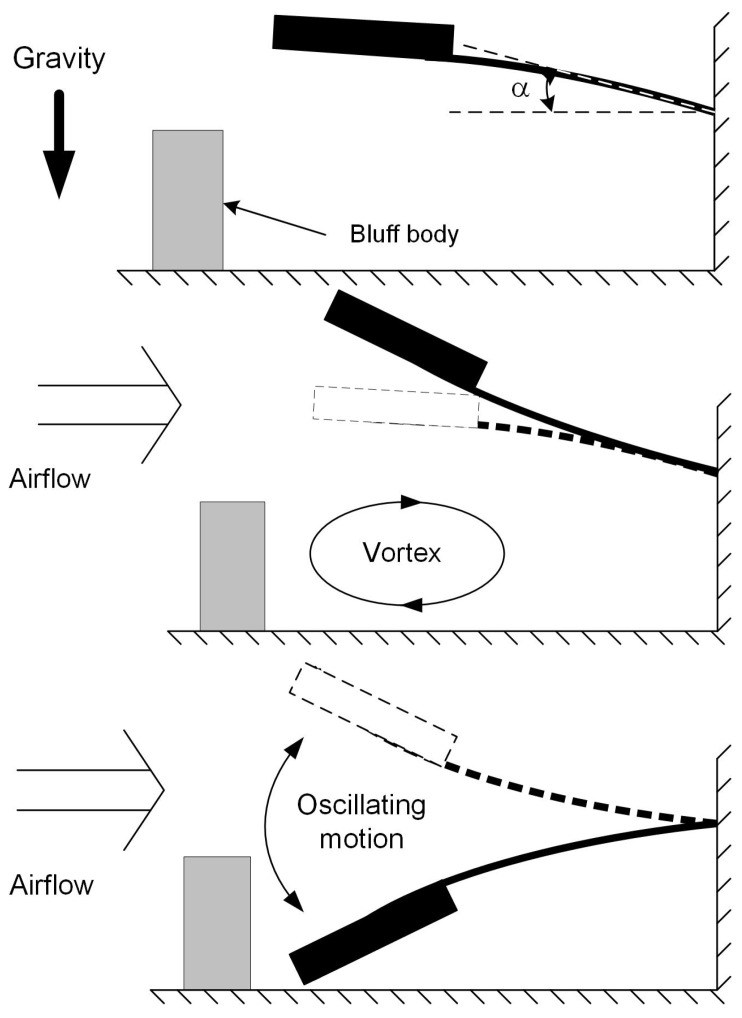
Operational principle of the flapping energy harvester [20]: (**top**) no airflow, initial downward bending due to the gravity; (**middle**) cantilever beam bent due to air flowing; (**bottom**) cantilever beam sprung back.

**Figure 2:**
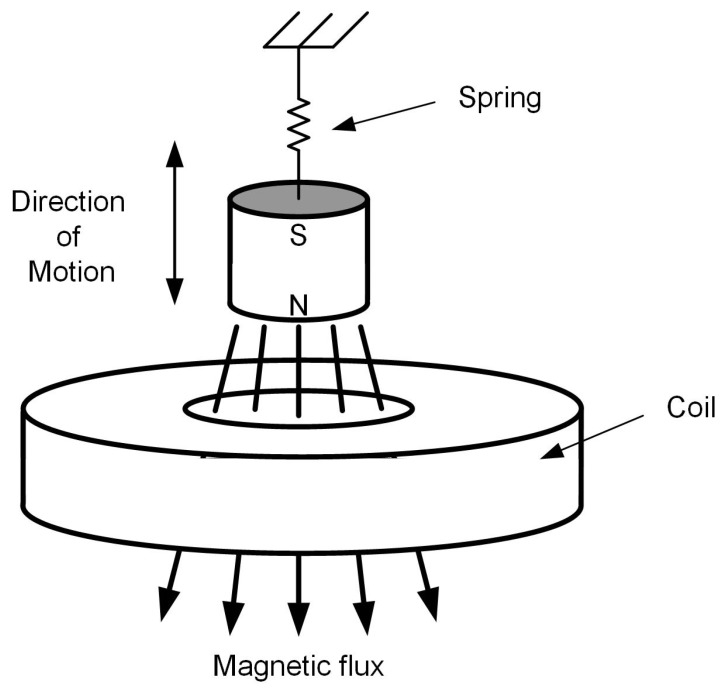
Electromagnetic transducer [20].

Based on previous work [20,21,22,23], the flapping airflow energy harvester investigated here is composed of a cantilever beam made of Beryllium copper, a wing attached to the free end of the cantilever with an attacking angle α of 10∼15${}^{\circ }$ (Figure 1), a bluff body placed in front of the wing with airflow coming towards it. As illustrated in Figure 1, the cantilever beam has an initial downward bending due to the gravity; it is lifted up by vortices induced by the bluff body and oscillates down under the joint effect of gravity and the vortices to form a cycle. An electromagnetic transducer (Figure 2) is implemented with the magnet part attached to the wing and the coil part mounted on the base. While the wing vibrates in the first mode, the attached magnet would oscillate along the centre line of the coil, producing a varying magnetic field dB which induces current in the coil. The generated power is determined by the gradient of magnetic field dB/dt which is directly related to velocity of the magnet, i.e., the displacement amplitude of magnet, *X*, as well as the oscillation frequency, *f*. Suppose the device works at a frequency close to its resonant frequency, the extracted power is mainly determined by the magnet velocity, proportional to the amplitude *X* which then becomes a key element to the increase of power generation and will be discussed in the next section. The reported problem of a pure resistive circuit has been encountered in the device introduced in this study. The electrical damping induced by electromagnetic coupling would gradually increase as the loading resistance decreases, which causes the system to stop oscillating before being tuned to the optimal resistive load or to oscillate in the second mode, thus being unable to generate power. In this study, in order to cope with this problem, mechanical interventions are considered. Both flow velocities and application of external magnetic excitation were investigated regarding their effect on aiding the oscillation of the device and on enhancing power output. All related geometric and physical properties of this device are given in Table 1.

## 3. Effect of Damping Ratio on Magnification for a System of One Degree of Freedom

As mentioned in the previous section, the amplitude of oscillation *X* is a key element to enhance power output when the device works at a frequency close to its natural frequency. This section will focus on analysis of *X* and identification of related factors affecting its value. As the vibration in the second mode only happens at relatively high wind velocity (>7 m·s${}^{-1}$, Re > 24,000) and high damping ratio (total damping ratio ζ>0.02) when the device has stopped working, this system will be approximated to one with one degree of freedom to facilitate the analysis of a magnification factor.

The general motion equation of a forced system with one degree of freedom is given by [24]

(1) $$m\frac{d^{2}x}{dt^{2}}+c\frac{dx}{dt}+kx=F,$$

where *m* is the mass of the moving body, *c* is the system’s damping factor, *k* is the spring constant and *F*, the forcing function, is the applied force which is supposed to be harmonic in this study and has the form: $F=F_{0}\cos\omega t$ with $F_{0}$ the amplitude and ω the angular frequency. As the transient response fades away, the displacement amplitude, *X*, under this harmonic oscillation is given by [24]

(2) $$X=\frac{F_{0}}{{\left[{\left(k-m\omega^{2}\right)}^{2}+c^{2}\omega^{2}\right]}^{1/2}}.$$

**Figure 3:**
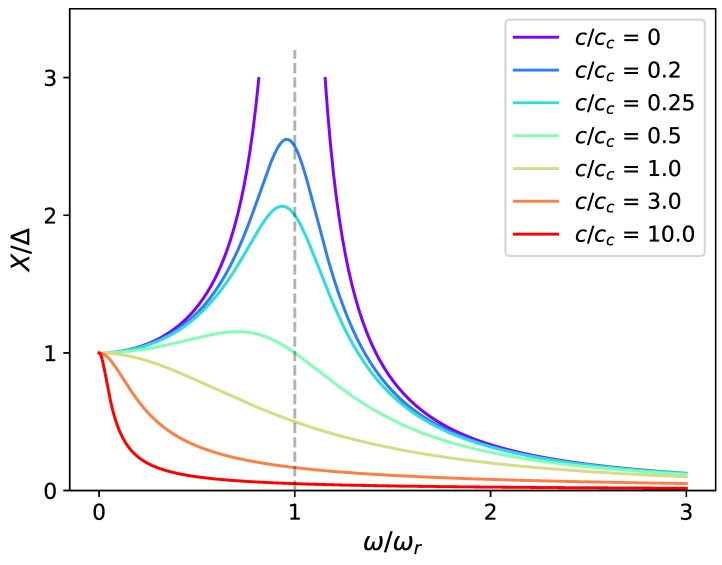
Dynamic magnification factor X/Δ against $\omega /\omega_{r}$ for different damping ratios $\zeta =c/c_{c}$.

**Figure 4:**
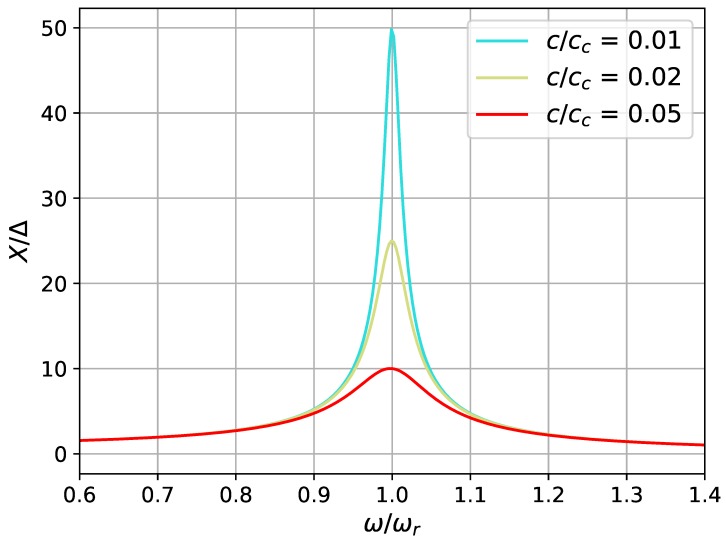
Magnification factor X/Δ for small damping ratios from 0.01 to 0.05.

**Figure 5:**
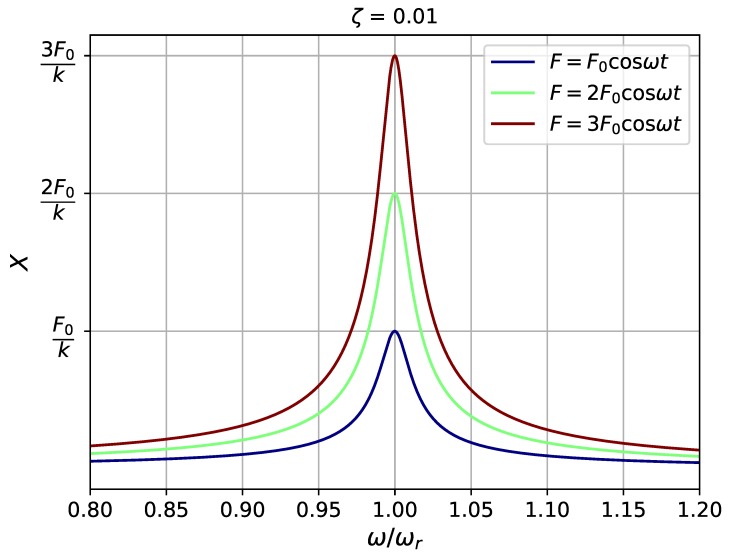
Amplitude of displacement *X* against $\omega /\omega_{r}$ for different damping ratios $c/c_{c}$.

The magnification factor is then defined as the amplitude *X* normalized by the displacement of the mass under a static force, $\Delta =F_{0}/k$:

(3) $$\frac{X}{\Delta }=\frac{1}{{\left\{{\left[1-{\left(\omega /\omega_{r}\right)}^{2}\right]}^{2}+4{\left(c/c_{c}\right)}^{2}{\left(\omega /\omega_{r}\right)}^{2}\right\}}^{1/2}},$$

where $\omega_{r}=\sqrt{k/m}$ is the resonant frequency, and $c_{c}=2\sqrt{km}$ is the critical camping coefficient. From the definition equation, it can be found that the magnification factor is determined by both the damping ratio ($\zeta =c/c_{c}$) and the frequency ratio ($\omega /\omega_{r}$). Of these two variables, the magnification factor X/Δ is very sensitive to the variation of damping ratio (Figure 3), which means the ability of an applied force to drive the system can be largely influenced by its damping condition. In addition, when the damping ratio is less than 1, small working frequencies are preferred, different from what is usually perceived that a system working at its resonant frequency has the largest amplitude of displacement. The total damping ratio, ζ, in this study is composed of two parts—the mechanical part and the electrical one. The mechanical damping ratio, $\zeta_{m}$, is the damping ratio measured in open circuit conditio when no electrical damping is added [25]. When the device is connected to an electrical load, additional electrical damping effect would be applied to the structure due to electromagnetic coupling. In this case, the measured damping is the sum of both mechanical and electrical damping. According to the measurements [25], the total damping ratio of the flapping airflow energy harvester is between 0.007 and 0.046. According to Figure 4, the system should achieve the maximum amplitude of displacement around its natural frequency. When the damping ratio is raised from 0.01 to 0.05, the magnification factor is reduced by 80% at the resonant frequency, which partially explains the behaviour of the device when the damping ratio is being tuned to its optimum (ζ increasing from less than 0.01 to approximately 0.04). Although the magnification factor is independent of the driving force, the absolute magnification amplitude is proportional to it as shown in Figure 5. When the magnification factor is largely reduced under a high damping ratio, one can still increase the applied force to increase the absolute magnification amplitude *X*. Two mechanical interventions will be investigated in the following to increase the applied force—the variation of flow velocities and exertion of external magnetic excitation.

## 4. Mechanical Interventions

### 4.1. Mechanical Intervention—Investigation of Airflow Velocities

**Figure 6:**
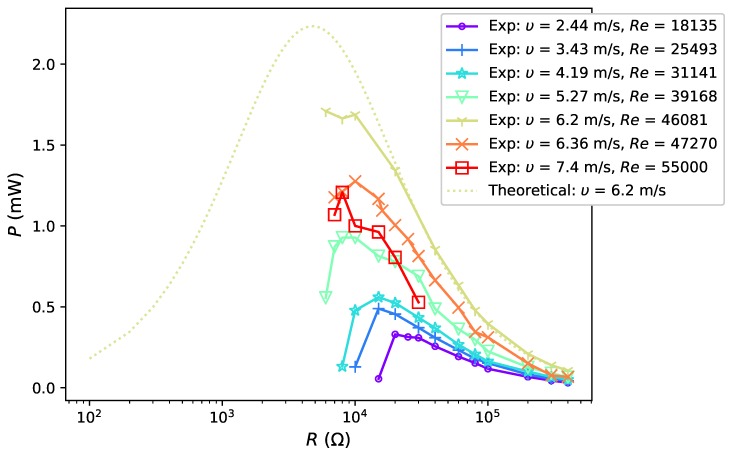
Power output under various flow velocities.

As the vortices to exert the applied force are directly associated with its velocity, the effect of airflow velocities on power output will be studied experimentally in this section. The harvester was placed in a uniform flow passing from its front with velocities, respectively, of 2.44 m·s${}^{-1}$, 3.43 m·s${}^{-1}$, 4.19 m·s${}^{-1}$, 5.27 m·s${}^{-1}$, 6.2 m·s${}^{-1}$, 6.36 m·s${}^{-1}$ and 7.4 m·s${}^{-1}$, corresponding to Reynolds number (Re) of 7914, 11,124, 13,589, 17,091, 20,108, 20,627 and 24,000, respectively. The voltage output was measured under open circuit condition, and with the loading resistance decreasing from 400 kΩ to a certain value when the device stopped oscillating due to excessive electrical damping. The airflow was held constant during this process. The resonant frequency of the system is 4.11 Hz according to simulation results and the actual working frequency varies from 3.4 Hz to 3.8 Hz, relatively close to the theoretical result. Thus, the power is considered to be generated when the oscillation amplitude of the device is well magnified and the results tested in these seven flow conditions are shown in Figure 6.

According to the results, higher flow velocity generally contributes to more power output as it has the potential to deliver more power to the energy harvester. It is also worth noticing that, in an experiment with the specific prototype, the oscillation of the device becomes unstable when the flow velocity is more than 6.2 m·s${}^{-1}$. First, the flow conditions become more turbulent (approximately when Re > 20,000). The wing tends to vibrate in the second mode and the power can not be effectively generated; secondly, the falling vortex, of which the frequency and intensity are related to the incoming velocity, moves downstream. However, as the wing is placed close to the bluff body, it is unable to benefit from the vortex street and the power output was reduced. As in practice, it is difficult to adjust the position of the wing, and research work from this point will focus on flow conditions with Re < 50,000 to investigate the relationship between flow velocity and power output. Figure 6 shows that, in all cases, output power drops sharply as the electrical load reduces below the optimum value. Furthermore, the load where the harvester stops oscillating decreases with the increase of flow velocity. To understand the second finding, attention was paid to the classical power generation equation of a system connected with a pure resistive circuit as [26]:

(4) $$P_{e}=\frac{V^{2}R_{load}}{{\left(R_{load}+R_{int}\right)}^{2}},$$

where $P_{e}$ is the generated electrical power, *V* is the voltage, $R_{load}$ is the load resistance and $R_{int}$ is the internal impedance of the system. According to maximum power transfer theorem [26], an optimal resistive load which maximizes the power output is equal to the internal resistance of the system, which is 4.77 kΩ in this case.

Theoretical power estimation is plotted in Figure 6 (yellow dotted line) based on Equation (4), with *V* equal to experimental voltage output measured in open circuit condition, $V_{OC}$, in an airflow of 6.2 m·s${}^{-1}$. As the load resistance decreases, this theoretical curve first continuously increases to the maximum and then gradually reduces to nearly zero. Ideally, experimental power output would follow the theoretical estimation. It would first increase to the maximum at $R_{int}$ and then gradually increase to zero as the load resistance decreases. Experimental output power in airflow of 6.2 m·s${}^{-1}$ has relatively good agreement with the experimental results when the load resistance decreases from 400 kΩ to 20 kΩ. However, as the load continues to decrease, the experimental power increase slows down and has slight drop when the load reaches 10 kΩ, and the device stops vibrating at a load less than 7 kΩ. In an airflow of 6.2 m·s${}^{-1}$, the minimal load to maintain the oscillation, $R_{min}^{m}$, is larger than $R_{int}$ and the harvester is unable to reach the theoretical optimal output power. In fact, when the load reduces to around 20 kΩ, the total damping ratio is increased to 0.019 and the amplitude magnification factor is reduced by 50%—from 50 to around 25. The force produced by the airflow is then insufficient to keep the device oscillating and the harvester would quickly stop oscillation as the damping ratio continues to increase, which explains that the actual power stops following the theoretical curve at 20 kΩ and sharply drops to zero.

As high speed airflow contributes more to the power output, it copes better with the increase of damping ratio compared to low speed airflow cases. The second finding is therefore explained: under the same damping ratio, the harvester can oscillate in a faster airflow while it can stop vibrating in a low speed condition, corresponding to the concept aforementioned—the absolute magnitude of displacement *X* can be enlarged by enhancing the applied force. To summarize, for flow conditions with Re up to 24,000 (wind velocity up to approximately 7 m·s${}^{-1}$), the power output increases with the flow velocity and the device can work at lower load resistances (i.e., higher damping) under higher velocities. In practice, one can place a funnel shaped structure in front of the bluff body to increase the velocity of airflow thus to improve the output power.

### 4.2. Mechanical Intervention—Application of External Magnetic Excitation

#### 4.2.1. Experimental Results and Discussion

**Figure 7:**
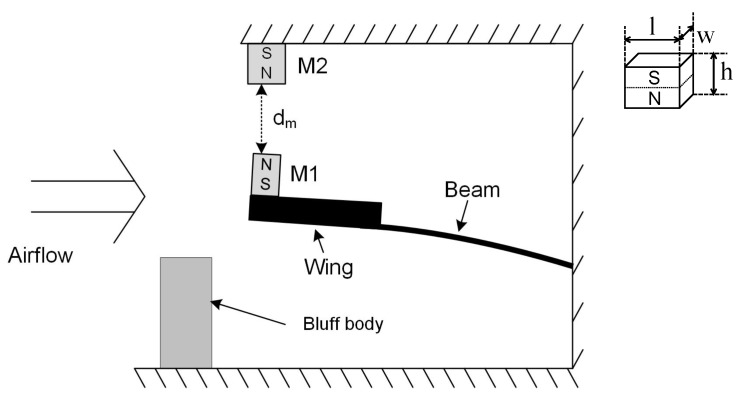
An external magnet placed above to aid cantilever oscillation.

**Figure 8:**
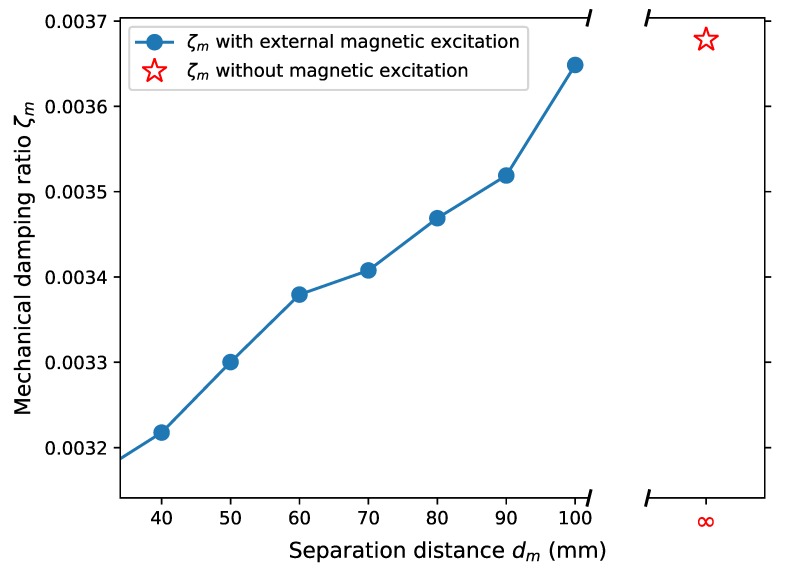
Mechanical damping ratio $\zeta_{m}$.

The second mechanical intervention for power improvement through increase of applied force is to apply external magnetic excitation. In order to achieve the excitation, a pair of neodymium magnets of dimension 15 mm (l) × 10 mm (h) × 5 mm (w) is applied (Figure 7). One magnet (M1) is placed on the wing of the harvester while the other (M2) is fixed right above it. Two magnets have identical poles facing each other to exert a repelling force when the wing approaches, with the magnetic field between two magnets, *B*, varying from approximately 0.13 to 0.47 T (simulation results). When the harvester oscillates upwards to its maximum displacement, it has additional downwards excitation force exerted by these two magnets, which aids the movement of the wing when the damping ratio becomes high. First, it is worth exploring the variation in mechanical damping ratio induced by application of external excitation. The mechanical damping ratio $\zeta_{m}$, measured in an open circuit condition, is shown in Figure 8. The damping ratio is slightly reduced by approximately 0.0005 with a relatively strong excitation (a separation distance of 40 mm), which is negligible as the minimal total damping ratio is approximately 0.008. Thus, the potential change in power output is unlikely to be affected by the mechanical damping ratio change caused by the additional magnets.

**Figure 9:**
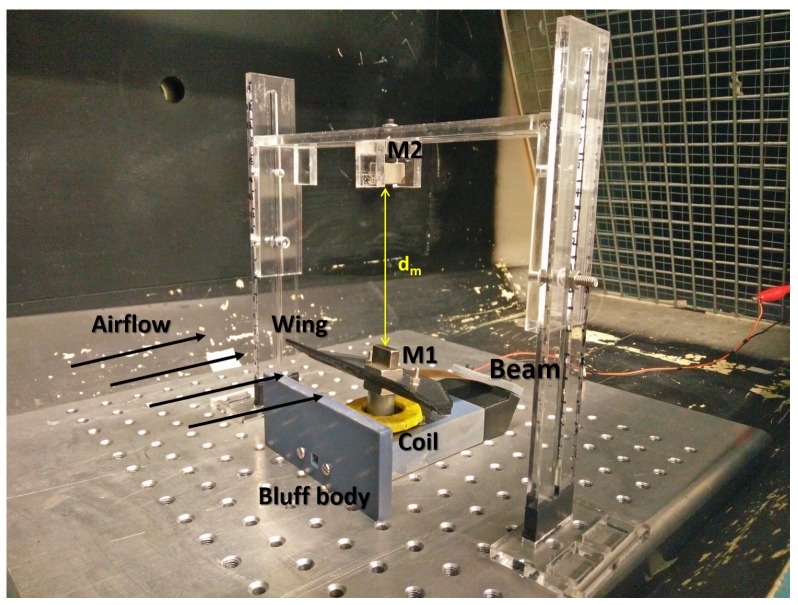
The experimental setup for investigation of magnetic excitation.

Three variables are investigated to evaluate the effectiveness of this intervention. The first one is the separation distance between magnets, $d_{m}$, which serves as a local scale to help understand the strength of magnetic excitation under the same circumstances; the second is the root mean square output voltage measured in open circuit conditions ($V_{OC}$), to evaluate the influence exerted on the amplitude of displacement by external excitation; the third is the power output, $P_{e}$, acquired based on voltage measurement under various resistive loads, to evaluate the effectiveness of magnetic excitation in improving power output. Similar to the tests in different airflows, the device was placed in the middle of a wind tunnel with additional excitation implemented as shown in Figure 9.

**Figure 10:**
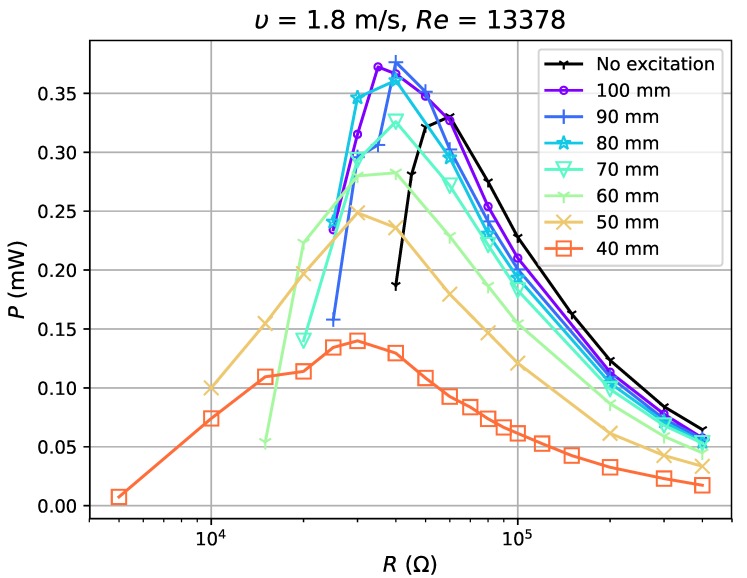
Power output for excitation application at 1.8 m·s${}^{-1}$.

**Figure 11:**
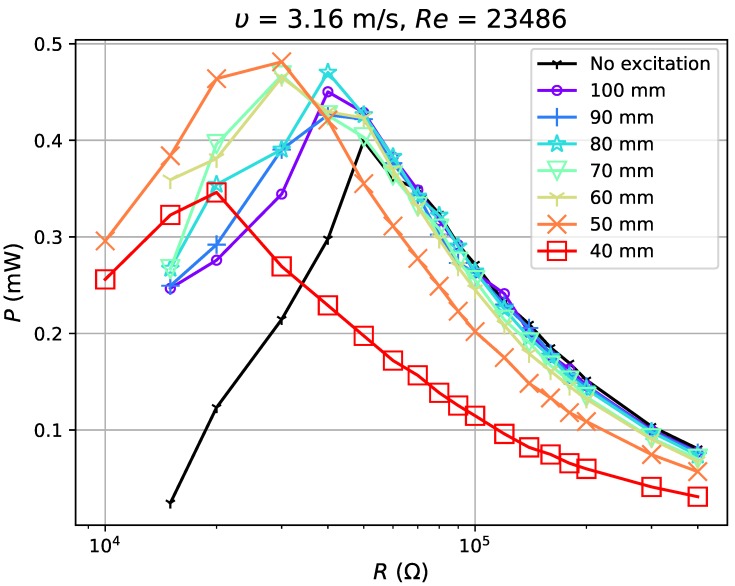
Power output for excitation application at 3.16 m·s${}^{-1}$.

**Figure 12:**
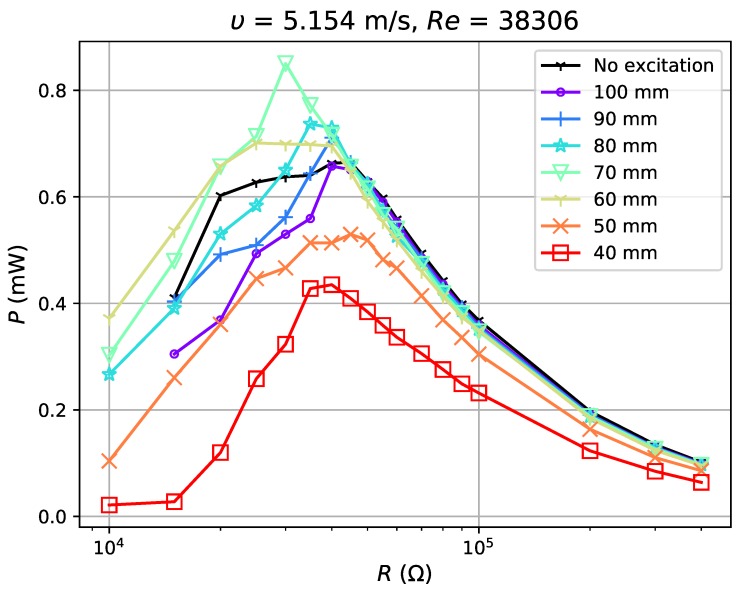
Power output for excitation application at 5.154 m·s${}^{-1}$.

**Figure 13:**
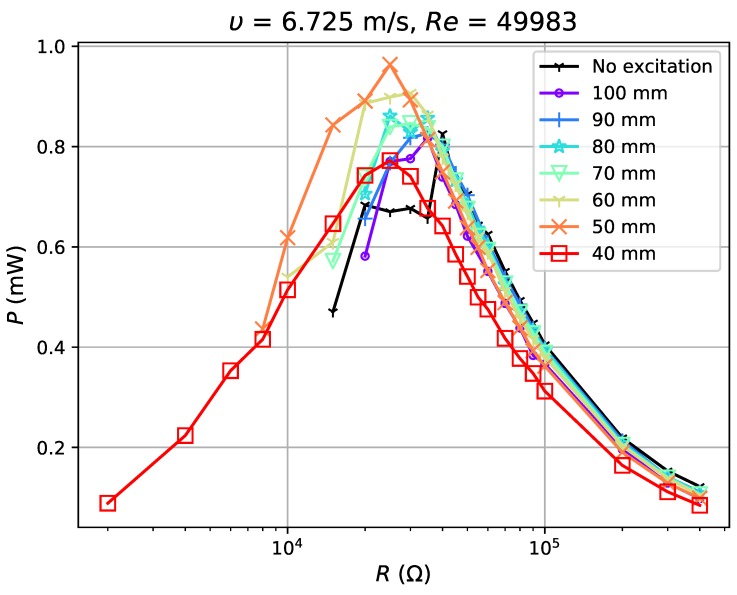
Power output for excitation application at 6.725 m·s${}^{-1}$

In the test, airflow varied from 1.8 m·s${}^{-1}$ (Re=5837) to 6.725 m·s${}^{-1}$ (Re=21,810). For each flow condition, the separation distance $d_{m}$ was gradually adjusted from 40 mm to 100 mm with a step of 10 mm (a weakening excitation). The experimental results show reduction in the voltage ratio, $\gamma_{V}$, of open circuit voltage with excitation, $V_{OCM}$, to that without excitation, $V_{OCN}$ ($\gamma_{V}=V_{OCM}/V_{OCN}$) by 52–99% with the application of magnets. The power outputs tested in four flow conditions (Figure 10, Figure 11, Figure 12 and Figure 13) show, however, an increase when the magnetic excitation is properly integrated. The results will be explored in more detail to understand how this intervention is able to increase output power. Take the flow velocity of 1.8 m·s${}^{-1}$, for example. Figure 10 contains eight cases, one without magnetic excitation and the other seven with $d_{m}$ increasing from 40 mm to 100 mm, implying a decrease in applied excitation. When compared with the case without excitation, the application of a large excitation ($d_{m}=40$ mm) has widened the effective load range of the harvester, but, at the same time, reduces the maximum power output by approximately 60%. As the separation distance gradually increases to 70 mm, the excitation is weakened and the power output begins to equal that of the case without a magnet. In this case, the minimum load that maintains the oscillation, $R_{min}^{m}$, is 15 kΩ, which is larger than the value of 5 kΩ at d=40 mm, but still smaller than the no excitation case (40 kΩ). As $d_{m}$ continues to increase to 90 mm, the power output begins to surpass the case without magnets by a maximum of 12.7%. It drops slightly when the distance is raised up to 100 mm. Apart from certain instability, the experiments in 3.16 m·s${}^{-1}$, 5.154 m·s${}^{-1}$ and 6.725 m·s${}^{-1}$ airflows have similar results: the power output of the harvester is reduced under large external excitation ($d_{m}=40$ mm) and gradually increases as the excitation weakens; the power surpasses that of the no excitation case when $d_{m}\geq 60∼70$ mm and reaches a peak when $d_{m}=80$ or 90 mm and the power can be increased by up to 30%. As the distance increases to 100 mm, the power output becomes approximate to that of the no excitation case, which is equivalent to the condition when the separation distance between magnets is infinite. By observing all cases with better power output, it is noticed that they all have $R_{min}^{m}$ at a moderate value and slightly reduced $V_{OC}$. If the magnetic excitation is too strong and significantly influences the generated voltage, even a small $R_{min}^{m}$ is not sufficient to compensate for the reduction in power output. Therefore, the excitation needs to be properly applied so that the voltage is mildly restrained, guaranteeing that the power gap between excitation and no excitation cases can be compensated. In such a condition, with a decreased $R_{min}^{m}$, the energy harvester is able to keep its oscillation at a damping ratio where it would have stopped oscillating in the no excitation case, and thus generates more power. The output power of the harvester is improved through the balance achieved between the decrease in $R_{min}^{m}$ and reduction in $V_{OC}$.

#### 4.2.2. Evaluation of the Mechanical Intervention in Power Increase

**Figure 14:**
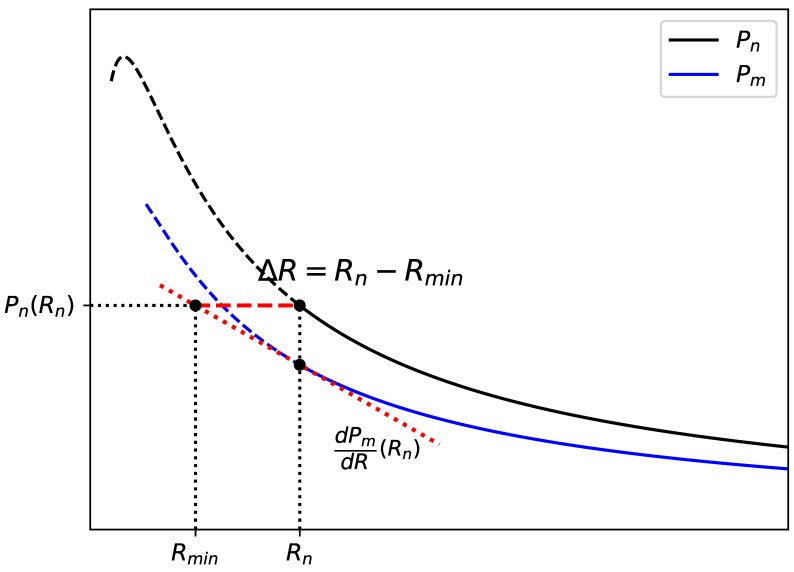
The minimal resistance difference required for power increase.

In consideration of the balance between the decrease in $R_{min}^{m}$ and reduction in voltage output, the effectiveness of the mechanical intervention in power improvement is discussed in this section based on voltages measured in open circuit condition with and without magnetic excitation ($V_{OCM}$ and $V_{OCN}$). The classical power output with a pure resistance (Equation (4)) is illustrated in Figure 14 to help understand how the the balance between voltage reduction and decrease in minimal load increases the power. Figure 14 illustrates two curves, $P_{m}$ and $P_{n}$, theoretical power output of cases with and without magnetic excitation calculated based on Equation (4), respectively. $R_{n}$ is the optimal resistance which maximizes $P_{n}$ with the maximal power to be $P_{n}\left(R_{n}\right)$. Experimentally, the device would soon stop oscillating as the loading resistance continues to decrease to $R_{min}^{m}$. The power gap between two curves at $R_{n}$ is

(5) $$\Delta P=P_{n}\left(R_{n}\right)-P_{m}\left(R_{n}\right)=\left(V_{n}^{2}-V_{m}^{2}\right)\frac{R_{n}}{{\left(R_{n}+R_{int}\right)}^{2}},$$

where $V_{n}$ and $V_{m}$ are voltages for cases with and without magnetic excitation, respectively, corresponding to experimental measurements $V_{OCN}$ and $V_{OCM}$. Suppose $P_{m}$ will continue to augment by the derivative from the point $\left(R_{n},\;P_{m}\left(R_{n}\right)\right)$, which is given by

(6) $$\left|\frac{dP_{m}}{dR}\left(R_{n}\right)\right|=V_{m}^{2}\frac{R_{n}-R_{int}}{{\left(R_{n}+R_{int}\right)}^{3}}.$$

Then, the minimal load variation, ΔR, to compensate the power difference ΔP is given as:

(7) $$\Delta R=\left(\frac{1}{\gamma_{V}^{2}}-1\right)\frac{R_{n}\left(R_{n}+R_{int}\right)}{R_{n}-R_{int}}$$

with the voltage ratio $\gamma_{V}=V_{m}/V_{n}$. As the optimal load $R_{n}$ and internal resistance $R_{int}$ are constant for a certain airflow, the required minimal ΔR is only dependent on the voltage ratio and is inversely proportional to it, which explained the balance observed in the experiment for power increase. As in the experiment, the device may quickly stop oscillation due to a high damping ratio, interventions with a small required load difference ΔR and a relatively high voltage ratio can help achieve a power increase. In this study, the optimal load $R_{n}$ of the no excitation case is much larger than the internal resistance $R_{int}$ and thus ΔR can be approximated to

(8) $$\Delta R\approx \left(\frac{1}{\gamma_{V}^{2}}-1\right)R_{n}.$$

**Figure 15:**
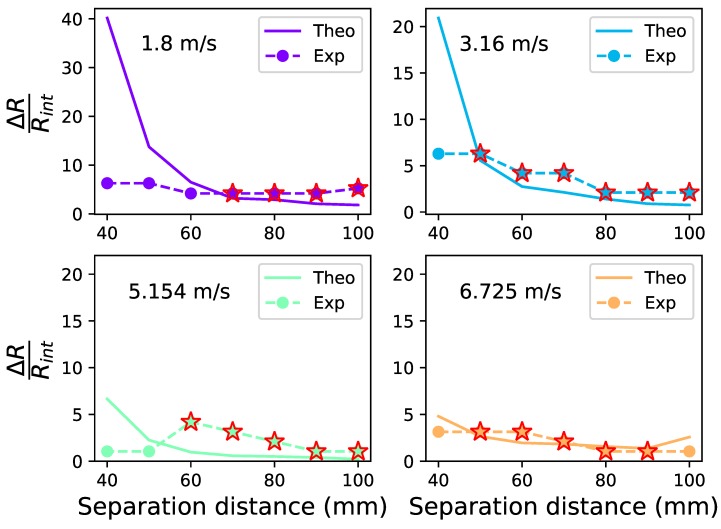
Theoretically predicted and experimental range factors.

This required minimum load difference ΔR is normalized by being divided by the internal resistance $R_{int}$. The ratio $\gamma_{R}=\Delta R/R_{int}$ is taken as a load variation factor and can be predicted based on $V_{OCM}$ and $V_{OCN}$ with $\gamma_{V}=V_{OCM}/V_{OCN}$. The experimental $\gamma_{R}$ needs to at least surpass theoretical results so that the intervention may enhance power output.The comparison between two results are illustrated in Figure 15 where the solid lines are theoretical estimations, the dashed ones are experimental results and stars represent the cases which have generated power output more than the no excitation case. Results show that cases that enhance power generally have a load variation factor $\gamma_{R}$ larger than theoretical estimation and the theoretical values $\gamma_{R}$ of these cases are from 0.21 to 5.56.

## 5. Conclusions

This paper presents mechanical intervention methods to enhance the performance of a cantilever-based flapping airflow energy harvester by increasing flow velocity and using external magnetic excitation. The device consists of a clamped beam with a wing attached on its free end and a rectangular bluff body placed in front of the wing. This structure can stop oscillating by high electrical damping. In order to overcome this problem, one solution is to increase the input flow velocity and the other solution is to introduce external magnetic excitation. The magnetic excitation can be applied by implementing a magnet on the wing and another one above it to generate a repelling force during the oscillation of the wing, aiding the device to maintain oscillation even with a relatively high damping ratio. Both solutions are investigated experimentally. The case without excitation was first analysed and high airflow velocity was found to contribute to power increase. Experimental results showed that the output power was increased by approximately six times when the airflow velocity was increased from 2.44 m·s${}^{-1}$ to 6.2 m·s${}^{-1}$. Experiments with application of different magnetic excitation were run in airflow from 1.8 m·s${}^{-1}$ to 6.725 m·s${}^{-1}$ (Re from 7914 to 24,000). The results show that the power can be enhanced by up to 30% when the external magnetic excitation is properly integrated. If such excitation cannot provide sufficient repelling force to overcome the high damping force, it will not be able to improve the output power. On the other hand, if the repelling force exerted by the external excitation exceeds the ideal value, it will introduce extra damping which reduces the output power. Therefore, careful consideration must be taken when this method is adopted in real world applications.

## Figures and Tables

**Table 1:** Geometric and physical properties of the cantilever-based flapping energy harvester.

Parameter	Description	Value
*E* (Pa)	Young’s modulus of the beam	$125\times 10^{9}$
*L* (m)	Length of the beam	0.062
*w* (m)	Width of the beam	0.018
*h* (m)	Thickness of the beam	0.0003
*m* (kg)	Mass of the wing with magnet (tip mass)	0.0698
$L_{w}$ (m)	Span of the wing	0.11
$w_{w}$ (m)	Chord of the wing	0.048
α (${}^{\circ }$)	Attack angle of the wing in experiment	10∼15
$R_{int}$ (Ω)	Internal resistance of the system	4770
ν (m${}^{2}\cdot$s${}^{-1}$)	Kinematic viscosity of air at 15${}^{\circ }$	$1.48\times 10^{-5}$
$\zeta_{m}$	Mechanical damping without magnetic excitation	0.0037
